# Drinking and Social Media Use Among Workers During COVID-19 Pandemic Restrictions: Five-Wave Longitudinal Study

**DOI:** 10.2196/33125

**Published:** 2021-12-02

**Authors:** Atte Oksanen, Reetta Oksa, Nina Savela, Magdalena Celuch, Iina Savolainen

**Affiliations:** 1 Tampere University Faculty of Social Sciences Tampere Finland

**Keywords:** excessive drinking, alcohol, COVID-19, social media, remote work, psychological distress, distress, pattern, trend, prediction, survey, app, risk

## Abstract

**Background:**

The COVID-19 pandemic restricted everyday life during 2020-2021 for many people worldwide. It also affected alcohol consumption patterns and leisure activities, including the use of social media.

**Objective:**

The aim of this study was to analyze whether social media use predicts increased risky drinking over time and during the COVID-19 pandemic restrictions in particular.

**Methods:**

This 5-wave longitudinal survey study, based on a nationwide sample of workers, was conducted in Finland in 2019-2021. A total of 840 respondents (male: 473/840, 56.31%; age range 18-64 years; mean age 43.90, SD 11.14 years) participated in all 5 waves of the study. The outcome variable was risky drinking, measured using the 3-item Alcohol Use Disorders Identification Test (AUDIT-C). Multilevel linear hybrid modeling enabled the investigation of both within-person and between-person effects. Predictors included social media use and communication, involvement in social media identity bubbles, psychological distress, and remote working. Controls included sociodemographic factors and the Big Five personality traits.

**Results:**

Increased involvement in social media identity bubbles was associated with an increase in risky drinking behavior. Of all social media platforms examined, online dating app use was associated with riskier use of alcohol over time during the COVID-19 crisis. Daily social media communication with colleagues about nonwork topics was associated with risky drinking. Female gender, younger age, university education, nonindustrial occupational field, conscientiousness, agreeableness, and neuroticism were associated with lower levels of risky drinking.

**Conclusions:**

Social media use during a pandemic carries some risks for alcohol consumption. Involvement in social media identity bubbles and online dating are risk factors for excessive drinking during the COVID-19 pandemic.

## Introduction

The COVID-19 pandemic has remarkably changed everyday life for people worldwide. Restrictions placed on social gatherings, public events, bars, and restaurants are likely to affect peoples’ drinking habits [1,2]. Researchers have argued that the COVID-19 crisis has put home drinking in focus more than ever before [3,4]. Recent studies have reported increased drinking during the COVID-19 pandemic [5-8]; however, few cross-sectional studies have reported both a decrease as well as no change in drinking [9-11]. Thus far, only a few studies have been based on longitudinal samples [1,6,7,12,13]. Additionally, it seems that individuals react to the COVID-19 crisis differently, and those facing emotional worry and distress are especially at risk for increased drinking [1,9,14-18]. There might be specific risk factors associated with increased drinking during the COVID-19 pandemic, and the role of social media in drinking, in particular, calls for attention.

Different forms of social media have become a central part of communication and interaction among people across the world [19-21]. Social media has evolved through a transformation from Web 1.0 society to Web 2.0 society, with web-based global services enabling individuals, communities, and organizations to engage, produce, co-create, modify, and share individualized user-generated content, as well as interact, collaborate, and manage their social networks on the internet [21,22]. Social media has numerous advantages, such as quick transmission of information and fostering social connectedness [23,24]. However, social media use also has downsides, and previous research has established a relationship between social media use and higher alcohol consumption [25,26], which has been investigated particularly among young users and college students [27-33]. Furthermore, it seems that some social media apps are more popular among risk drinkers. For example, studies on Tinder users have shown that they drink more than nonusers do [34-36]. Dating app users are also more likely to engage in hookups involving alcohol use [37,38].

The underlying mechanisms between social media use and related alcohol use are not entirely known, although previous studies suggest that perceived drinking norms and exposure to alcohol content and marketing might be possible factors [25,39-41]. A meta-analysis showed that exposure to and engagement with alcohol-related content on social media were associated with higher self-reported drinking and alcohol problems among adolescents and young adults [42]. Individuals’ attitudes and positive alcohol-related cognitions are motivational determinants of alcohol use [39]. Moreover, social media has been argued to be a source for social influence altering peer-drinking norms [32]. Drinking-related content on social media has become common for some users during the pandemic, and it has potential to lead to increased drinking [43].

Research on social media identity bubbles (ie, social cliques) shows that people are drawn toward similar-minded individuals with whom they identify strongly and share information [21,44,45]. Thus far, identity bubbles based on drinking during the COVID-19 pandemic have not been investigated. It is likely that in the absence of regular social activities and places to drink, people engage in drinking while interacting with their friends and colleagues on the internet. Social media is known to induce the development and duration of web-based social drinking events for normal and risky drinkers, enabling large gatherings of groups regardless of their location and prior familiarity [25]. Social media apps may promote users to find other similar-minded individuals to drink with at private parties, although this practice is not encouraged by health officials during the pandemic [46].

There are likely to be major differences in drinking during the COVID-19 pandemic at the individual level. Although the ongoing pandemic has been reported to have a negative impact on the mental well-being of people in Europe [47,48], it seems that mental health of the Finnish working population did not decline dramatically during the COVID-19 crisis, probably due to the relatively mild course of the pandemic in Finland [49]. Nevertheless, previous studies have shown that, for many people, lockdowns and changes in work conditions, with the expansion of remote work, have been associated with increased psychological distress [1,24,50,51]. In turn, psychological distress has been linked to an upsurge in alcohol use [1,52,53].

According to pre-pandemic studies, younger people and men are more likely to be risky drinkers [54-56]. Some studies have found similar connections during the COVID-19 pandemic for men [8,12,17,57] and people under 30 years of age [1]. However, female gender and younger age have been associated with both an increase and decrease in drinking [5,8,9,17,58]. The rise in drinking also varies across industries; for example, business, communication, and technology sector employees, as well as public administration employees, have reported increased drinking [1]. Higher education has been linked with increased drinking [58]. Poor financial situation has been associated with increased drinking [16,59], but also with reduced drinking [9]. A European study found that changes in drinking patterns were associated with financial stress, particularly among high-income groups—a decrease in drinking was observed among those reporting no financial stress and an increase in drinking was noted among those reporting financial distress [10]. People with conscientious personality characteristics were less likely to increase drinking during the COVID-19 crisis [1].

This longitudinal study focused on analyzing risky drinking behaviors before and during the COVID-19 pandemic restrictions in Finland. The first COVID-19 case in Finland was reported in January 2020, and the country reacted to the rising worldwide crisis in March 2020 by placing several restrictions: large public events were cancelled and recommendations for remote work were implemented [60]. On March 16, 2020, a national state of emergency was declared. Following the new emergency legislation, bars and nightclubs were forced to close, and restaurants were only allowed to sell food and low-alcohol beverages (eg, beer) to go. Finland does not allow alcohol to be delivered to homes, but monopoly stores and supermarkets were open as usual. The state of emergency was lifted in mid-June 2020; however, new restrictions were implemented again in October 2020. These restrictions concerned large public events, limiting the amount of people allowed to gather and the hours of operation of bars, nightclubs, and restaurants. The emergence of the new Delta variant of SARS-CoV-2 presented challenges to the existing guidelines. Subsequently, a state of emergency was declared again in the beginning of March 2021, leading to the closure of bars and nightclubs, and restaurants were only allowed to sell food and low-alcohol beverages to go. The second state of emergency lasted until April 27, 2021. The restrictions for bars, nightclubs, restaurants, and cultural events were gradually eased up during summer and autumn 2021. The recommendation for remote work was in place until October 15, 2021.

This longitudinal study aimed to analyze risky drinking behaviors before and during the COVID-19 pandemic restrictions in Finland. The research questions were (1) How did alcohol use change during the COVID-19 pandemic restrictions? (2) Do social media–related factors predict alcohol use over time? (3) Are psychological and social factors, such as psychological distress, remote working, and personality, associated with drinking?

## Methods

### Participants

The longitudinal *Social Media at Work in Finland Survey* study started in Finland in mid-March 2019, and data were collected at 6-month intervals. A total of 5 waves were collected from Finnish employees. The baseline survey data collection commenced in mid-March 2019 (time point 1 [T1]; N=1817), with a response rate of 28.3%. The same participants were contacted for the follow-up surveys in mid-September 2019 (T2; n=1318), mid-March 2020 (T3; n=1081), mid-September 2020 (T4; n=1152), and mid-March 2021 (n=1018). The third survey was sent only to those who had responded to the second survey, whereas the fourth and fifth surveys were sent to all original respondents. Of the original 1817 survey respondents, 840 (46.23%) participated in all 5 surveys.

The survey was designed to represent the Finnish working population. The survey was conducted in Finnish, and responses were collected from all areas of mainland Finland. All participants were working at T1, and they were from various major occupational fields (see Multimedia Appendix 1 for details). Comparison of T1 participants to official statistics on Finnish workers provided by Statistics Finland showed that data collection was successful. We found no major deviations in sociodemographic factors, including age, gender, and geographical area [61-63]. The sample was similar to the official census data in other ways as well. Although official census figures are not available for the working population only, the proportion of excessive drinking in our sample was close to national statistics data for the 20-64 age group (ie, 30.35% male and 19.59% female participants in our sample vs 39% men and 24% women in the national population) [64]. Among the Finnish population of the 16-89 years age group, 58% used Facebook (vs 79.31% in our T1 sample), 39% used Instagram (vs 52.01% in our T1 sample), and 13% used Twitter (24.49% in our T1 sample) [65]. These differences are explained by the fact that our T1 sample included only workers aged 18 to 65 years.

Nonresponse analysis between those who responded to all 5 study waves (n=840) and those who responded only to some waves (n=977) showed that the final sample included slightly older respondents and more male participants. However, we found no differences in other variables included in this study, such as risk drinking, between those who had participated in all 5 surveys and others. In other words, dropout from the survey is not associated with our main points of interest. Our final sample is very close to the official census figures provided by Statistics Finland in terms of mean age (43.90 years in our sample vs 41.81 years in the Finnish population), gender (56.31% male participants in our sample vs 51.85% in the Finnish population), and university-level education (47.74% in our sample vs 42.46% in the Finnish population) [62,63].

The study protocol was reviewed and accepted by the Academic Ethics Committee of the Tampere region (decision 90/2018). All participants agreed to voluntarily participate in the web-based surveys, and they were informed about the purpose of the study and data processing procedures. Data collection was carried out in collaboration with Norstat, a data solutions provider.

### Measures

#### Drinking

We used the 3-item Alcohol Use Disorders Identification Test (AUDIT-C) to measure risky drinking. AUDIT-C is a widely used screening tool for excessive drinking [66-68]. AUDIT-C is considered to perform almost as well as the full 8-item AUDIT as a screening tool for risky drinking [68]. Three items measure frequency of drinking, units of alcohol per drinking occasion, and the frequency of heavy drinking. Each question has multiple response options, with risk points from 0 to 4, and the scale ranges from 0 to 12. A higher score indicates a higher level of risk drinking. The scale showed good internal consistency among time points (T1: α=.75, T2: α=.73, T3: α=.76, T4: α=.76, and T5: α=.77; see Table 1 for details)*.* Our models used AUDIT-C as a continuous variable. We also report the proportion of excess users, using a cutoff of ≥6 points for men and ≥5 points for women [64].

**Table 1:** Descriptive statistics of the main study variables.

Outcome variable		T1	T2	T3	T4	T5
Risky drinking, AUDIT-C score (range 0-12), mean (SD)		3.83 (2.50)	3.80 (2.44)	3.75 (2.51)	3.73 (2.47)	3.59 (2.51)
**Continuous predictors (range 0-1), mean (SD)**						
	Psychological distress	0.36 (0.17)	0.34 (0.16)	0.34 (0.15)	0.34 (.15)	0.34 (0.16)
	Social media identity bubble	0.45 (0.17)	0.46 (0.17)	0.46 (0.17)	0.47 (.16)	0.46 (0.17)
**Categorical predictors (code 0/1), %**						
	Online dating app use (eg, Tinder)	10.12	9.17	9.88	8.81	9.76
	Daily social media communication with colleagues about nonwork topics	10.95	9.40	11.31	9.64	9.52
	Social media use several times a day	35.00	34.29	38.69	38.57	39.64
	Remote work (≥3 days a week)	5.00	4.88	10.48	22.62	30.71

#### Psychological Distress

We used the 12-item General Health Questionnaire (GHQ-12) to measure psychological distress [69-72]. All 12 items concerning general mood and psychological strain have 4 answer options, rated on a Likert scale, ranging from very positive (0) to negative (3). The total scale ranged from 0 to 36, where higher scores indicate higher psychological distress. This scale was transformed to a scale of 0 to 1. The internal consistency of the scale was *excellent* at all time points (T1: α=.92, T2: α=.90, T3: α=.90, T4: α=.91, and T5: α=.92).

#### Social Media Identity Bubble

We measured involvement in social media identity bubbles using the 6-item Identity Bubble Reinforcement Scale (IBRS-6) [44]. Involvement in identity bubbles concerns strong social identification, homophily, and reliance on information from others on social media. Respondents were asked to respond to statements such as “On social media, I prefer interacting with people who share similar interests with me,” with response options ranging from 1 (*does not describe me at all*) to 7 (*describes me completely*). The original scale ranged from 6 to 42, but we transformed it to a scale of 0 to 1. The internal consistency of the scale was *good* at all time points (T1: α=.84, T2: α=.83, T3: α=.83, T4: α=.83, and T5: α=.85).

#### Use of Social Media Platforms

The use of different social media platforms and apps was determined by asking the study participants questions about their general use of social media (“How often do you use the following social media platforms?”) and their use of social media for work purposes (“How often do you use the following social media platforms for work purposes?”). Both questions were followed by separate items for different social media apps and internet services, such as Facebook, Instagram, Twitter, YouTube, LinkedIn, Microsoft Teams, and “Dating apps” (eg, Tinder). Answer options included “I don’t use it,” “less than weekly,” “weekly,” “daily,” and “many times a day.” All commonly used apps were first analyzed separately by using dummy variables for weekly users. In the final models, we used a dummy variable for use of online dating apps (0=no use and 1=at least sometimes). Sensitivity analyses were performed for weekly and daily users of online dating apps. The models also included a variable for general social media use. This variable was combined from the most used social media platforms (Facebook, Instagram, Twitter, or YouTube), and the dummy variable was created on the basis of social media use several times a day (0=less often than several times a day and 1=several times a day).

#### Daily Social Media Communication With Colleagues About Nonwork Topics

We used a single-item measure for daily social media communication with colleagues about nonwork topics: “How often do you use social media to keep in touch with your colleagues or work community regarding nonwork-related matters?” Answer options included “I don’t use it,” “less than weekly,” “weekly,” “daily,” and “many times a day.” A dummy variable was created indicating those communicating with their colleagues informally at least once a day. The measure was previously validated with two Finnish cross-sectional samples [23,24].

#### Remote Work

We measured remote work with a question on whether respondents were working remotely. Those respondents who indicated that they were working remotely at least 3 days a week were considered remote workers in this study.

#### Control Variables

Control variables included gender, age, education (MA degree or higher), and occupational area (industrial sector vs others). Gender included options for male, female, and other gender. Very few participants selected *other gender*, and none were among those who had participated in all waves (n=840). Hence, gender was used as a dummy variable (0=male, 56.31%; 1=female, 43.69%). Information on participants’ education was obtained via a question including 7 categories (see Multimedia Appendix 1 for details). A dummy variable was created to indicate those who have a master’s degree or higher from a university (0=no, 75%; 1=yes, 25%). For occupational field, we used the list of International Standard Industrial Classification of All Economic Activities (see details about the 7 broader categories in Multimedia Appendix 1). We used a dummy variable indicating the industrial area of workers (0=no, 70%; 1=yes, 30%) as a control in our analysis.

We used the 15-item Big Five Inventory for personality [73]. All items had responses ranging from 1 to 7, leading to 5 scales ranging from 3 to 21. These were transformed to a scale of 0 to 1 for the models: openness (mean 0.70, SD 0.16), conscientiousness (mean 0.75, SD 0.15), extroversion (mean 0.64, SD 0.21), agreeableness (mean 0.69, SD 0.14), and neuroticism (mean 0.56, SD 0.17). Internal consistency of the traits varied from acceptable (openness: *α*=.69; conscientiousness α=.67; agreeableness: α=.56; and neuroticism: α=.71) to good (extroversion: α=.87).

### Statistical Analyses

Descriptive results of the study are reported in Table 1 and the text. We provide information about general changes in risky drinking during 2019-2021. The main analyses focus on longitudinal predictors of risky drinking using linear multilevel hybrid models. With hybrid models, it is possible to estimate the within-person effect of time-variant variables, while simultaneously considering the between-person effects. Hybrid models combine the strengths of random-effects and fixed-effects approaches and address their shortcomings [74,75]. We ran the hybrid models with the xthybrid command in Stata (version 16.1; StataCorp) [75].

In our models, all main time-varying variables had both within-person and between-person effects. Within-person effects show how changes in predictors over time are associated with the change in the outcome variable. Between-person variables show group differences between individuals. The models also included several between-person control variables.

We first focused on analyzing within-person and between-person effects of social media app use on risky drinking. We performed analyses on the use of such apps, as well as on weekly and daily use, when possible. The full model reporting our main findings included only those social media apps that were found relevant during this analysis. Different sensitivity analyses were performed, and we also checked interactions with COVID-19 time points (T3-T5) to determine whether some effects were stronger or different during the pandemic.

## Results

Descriptive results showed changes in risky drinking measured were relatively small during the COVID-19 time points examined (T3-T5) compared to the pre-pandemic time points (T1-T2). Drinking decreased slightly, especially at T5 (see Table 1). The proportion of excessive drinkers (AUDIT-C score ≥6 for men and AUDIT-C score ≥5 for women) decreased slightly, from 25.24% (212/840) at T1 to 23.57% (198/840) at T5. Among male participants, no change was reported at all between T1 and T5, with 30.87% (146/473) reporting excessive drinking at both time points. However, excessive drinking among women declined from 17.98% (66/367) at T1 to 14.17% (52/367) at T5.

Our results showed that the most used social media apps were not strongly associated with risky drinking (see Multimedia Appendix 2 for details). Weekly use of Yammer had a small within-person effect (B=0.22; SD 0.11; 95% CI 0.01, 0.46; *P*=.04). Use of online dating apps (eg, Tinder) was strongly associated with risky drinking in all models. In particular, between-person effects were strong for users in general (B=1.52; SD 0.34; 95% CI 0.85, 2.19; *P<*.001), weekly users (B=1.85; SD 0.49; 95% CI 0.88, 2.81; *P<*.001), and daily users (B=2.06; SD 0.80; 95% CI 0.50, 3.62; *P=*.02) in models adjusting for age and gender. Therefore, of all the different social media apps, online dating app use was included in our final model.

Analysis based on the full hybrid model showed that of the within-person predictors, only involvement in social media identity bubbles was significantly associated with risky drinking (B=0.32; *P*=.04; see Table 2 for details). We found robust effects on some between-person variables. Online dating app users were more engaged in risky drinking than nonusers were (B=1.33; *P*<.001). Additionally, those respondents who had daily social media communication with colleagues about nonwork topics drank more than others did (B=0.96; *P*=.005). Women (B=−1.35; *P*<.001) and those with an MA degree or higher from university drank less than others did (B=–0.41; *P*=.02). Older age was associated with higher risky drinking (B=0.02; *P*=.01). Workers in the industrial sector drank more than others did (B=0.40; *P=*.02). Lower conscientiousness (B=−1.41; *P*=.01), lower agreeableness (B=−1.55; *P=*.005), and lower neuroticism (B=−1.17; *P*=.04) were associated with higher drinking. In addition, the full model shows that participants drank less during the COVID-19 era than they previously did (B=–0.13; *P*<.001).

The last part of our analysis aimed at reviewing whether these within-person and between-person effects were stronger during the pandemic. This analysis showed that the within-person effect of Tinder use was observed during the pandemic (B=0.29; SD 0.12; 95% CI 0.05, 0.53; *P*=.02). Other relevant interactions with time were not observed.

**Table 2:** Multilevel linear hybrid regression model showing within-person and between-person effects on drinking.

		B	Robust SE (B)	95% CI	*P* value	
**Within-person variables**						
	Psychological distress	−0.02	0.17	−0.35 to 0.32	.92	
	Social media identity bubble	0.32	0.15	0.02 to 0.62	.04	
	Use of a dating app (eg, Tinder)	0.16	0.09	−0.02 to 0.34	.08	
	Use of social media several times a day	−0.01	0.05	−0.12 to 0.09	.82	
	Daily social media communication with colleagues about nonwork topics	−0.01	0.06	−0.13 to 0.11	.88	
	Remote work	−0.11	0.05	−0.21 to 0.00	.04	
**Between-person variables**						
	Psychological distress	0.56	0.71	−0.83 to 1.95	.43	
	Social media identity bubble	−0.28	0.65	−1.54 to 0.99	.67	
	Use of a dating app (eg, Tinder)	1.33	0.33	0.68 to 1.99	<.001	
	Use of social media several times a day	0.16	0.20	–0.23 to 0.56	.42	
	Daily social media communication with colleagues about nonwork topics	0.96	0.34	0.29 to 1.64	.005	
	Remote work (≥3 days a week)	−0.19	0.33	−0.83 to 0.45	.57	
**Controls**						
	Female	−1.35	0.16	−1.66 to −1.03	<.001	
	Age	0.02	0.01	0.00 to 0.03	.02	
	MA degree or higher	−0.41	0.17	−0.74 to –0.08	.02	
	Industrial sector	0.40	0.17	0.05 to 0.74	.02	
	Openness	0.00	0.52	–1.01 to 1.02	>.99	
	Conscientiousness	−1.41	0.57	−2.53 to −0.29	.01	
	Extroversion	0.73	0.45	–0.15 to 1.61	.10	
	Agreeableness	−1.55	0.55	−2.62 to −0.47	.005	
	Neuroticism	−1.17	0.56	−2.26 to −0.08	.04	

## Discussion

### Principal Results

This longitudinal, 5-wave study investigated changes in risky drinking in 2019-2021. Longitudinal analysis showed that stronger involvement in social media identity bubbles was associated with higher risky drinking over time. A within-person effect of online dating app use (eg, Tinder) was also found, but only during the COVID-19 pandemic. This finding suggests that unusual circumstances have perhaps led to unusual and risky drinking habits. Online dating app users also drank more than others. Moreover, we found that daily social media communication with colleagues about nonwork topics was associated with risky drinking. Altogether, our results suggest that people engaged in drinking as part of involvement in web-based social bubbles. Furthermore, as dating in bars, clubs, and restaurants was not possible during the COVID-19 pandemic, dating apps such as Tinder were considered an alternative. These dating activities were found to be associated with risky drinking, despite the closure of bars.

### Comparison With Prior Work

Previous cross-sectional studies conducted during the COVID-19 pandemic have reported decreased drinking, especially in Europe [10], but increased drinking has also been reported [5-8]. Our results indicating a slight decrease in risky drinking are not surprising, because during spring of 2020 and 2021, there were severe restrictions on bars and restaurants, limiting the possibilities for social drinking. Previous longitudinal studies have shown that drinking less is related to lower access to typical drinking locations [10,12,58].

Our finding on the within-person effect of involvement in social media identity bubbles offers an interesting contribution to the literature. Previous studies have especially considered social media postings as motivational determinants of alcohol use [39] and generally discussed exposure to drinking cues on social media websites [25,42]. Our findings expand this notion by considering the potential effect of engaging with similar-minded individuals. We further noted that individuals communicating with colleagues about nonwork-related issues tended to drink more. As our models controlled for personality, we believe these findings reveal something interesting about the role of social media in the context of drinking during the COVID-19 pandemic.

We discovered that increased use of dating apps was associated with increased risky drinking during the pandemic. Our longitudinal evidence supports previously noted findings that dating app users drink more than nonusers, and this pattern increases during their dates [34-36]. It is likely that alcohol is consumed during the dates, despite bars being closed. Our study contributes to the growing number of studies on risks and risky behavior related to dating apps [76,77].

Our findings further showed that female gender, younger age, university education, nonindustrial occupational field, conscientiousness, agreeableness, and neuroticism were associated with lower levels of drinking. These results reflect the findings of previous studies [1,58].

We found no longitudinal evidence on the effect of psychological distress on risky drinking during the COVID-19 pandemic. This finding contrasts previous findings on the role of psychological distress and mental well-being in drinking during the pandemic [9,14-18]. One potential reason for this might be that in global comparisons, the COVID-19 situation was manageable in Finland during 2020 and spring 2021. For example, a report by *Der Spiegel* indicated that Finland had coped the best with COVID-19 in comparison with 154 other countries [78]. It is feasible that spring 2020 only presented a major stress for people, reflecting increased drinking among those who were distressed [1]. It is, however, quite early to estimate potential long-lasting effects of the COVID-19 pandemic on drinking levels, as the crisis is still ongoing.

### Limitations and Strengths

Our study was limited to Finland and its working population. Hence, some risk drinking groups, including full-time students and retired elderly people, were not part of the study. The results only cover the timeframe of 2019-2021, and it is too early to estimate how the crisis and drinking will further develop. Our results are limited to self-reported information on drinking. Despite these limitations, a major strength of our study is the use of multi-wave, longitudinal data. Data collection started before the COVID-19 crisis, enhancing estimation of the effects of the pandemic on drinking. Our data are particularly strong on social media measures, and the response rate for surveys was high. Our modelling strategy used within-between person hybrid models that provide an advanced way of estimating longitudinal effects of independent variables.

### Conclusions

This longitudinal study of workers in Finland showed a decrease in risky drinking during the COVID-19 crisis. The main risk factors for increased risky drinking were found to be related to social media use. Involvement in social media identity bubbles was associated with risky drinking over time. Dating app users were more likely to drink more during the pandemic. Moreover, daily social media communication with colleagues about nonwork topics was associated with higher risky drinking. Our results provide significant evidence of the power of social media during unusual times.

The role of social media in drinking should be considered by social media companies such as Tinder to monitor and regulate alcohol-related postings and advertising on their services and promote more healthy ways of social interaction. This is especially essential during a crisis like the COVID-19 pandemic, when social media services present virtually the only possibilities for forming new social relationships due to lockdowns and restrictions on movement. The connection between social media use, involvement in social media identity bubbles, and increase in alcohol consumption are important findings for organizations and policy makers alike. Education and prevention efforts should consider these risk factors and, possibly, be targeted to at-risk groups.

## Appendix

Multimedia Appendix 1 Education and occupational area of the participants at T1 (n=840).

Multimedia Appendix 2 Descriptive statistics on use of social media platforms and associations with risky drinking in multilevel linear hybrid regression models.

## Abbreviations

AUDIT-C: 3-item Alcohol Use Disorders Identification Test
GHQ-12: General Health Questionnaire
IBRS: 6-item Identity Bubble Reinforcement Scale
