# Supplementary material for: Drinking and Social Media Use Among Workers During COVID-19 Pandemic Restrictions: Five-Wave Longitudinal Study
Source: J Med Internet Res. 2021 Dec 2;23(12):e33125. doi: 10.2196/33125 (PMC8641700; doi:10.2196/33125)
Supplement: Multimedia Appendix 1 [file jmir_v23i12e33125_app1.docx]

**Table S1.** Education and occupational area of the participants at T1 (n = 840)

|  | n | % |
| --- | --- | --- |
| **Education** |  |  |
| Primary education only | 24 | 2.86 |
| Upper secondary school degree | 82 | 9.76 |
| Vocational school degree | 203 | 24.17 |
| Degree from vocational college or similar | 130 | 15.48 |
| University of applied sciences degree (typically only bachelor) | 191 | 22.74 |
| University degree (typically master's degree) | 188 | 22.38 |
| Doctoral degree | 22 | 2.62 |
|  |  |  |
| **Occupational area** |  |  |
| Agriculture, forestry, and fishing; mining^1^ | 13 | 1.55 |
| Manufacturing^1^ | 102 | 12.14 |
| Electricity, gas, and heating supply; water supply; sewerage and waste management activities^1^ | 12 | 1.43 |
| Construction^1^ | 35 | 4.17 |
| Wholesale and retail trade; repair of motor vehicles and motorcycles^1^ | 51 | 6.07 |
| Transportation and storage^1^ | 39 | 4.64 |
| Accommodation and food service activities | 19 | 2.26 |
| Information and communication | 42 | 5.00 |
| Financial or insurance activities | 17 | 2.02 |
| Real estate activities | 19 | 2.26 |
| Professional, scientific, and technical activities | 41 | 4.88 |
| Administrative and support service activities | 16 | 1.90 |
| Public administration and defense; compulsory social security | 54 | 6.43 |
| Education | 83 | 9.88 |
| Health and social work activities | 135 | 16.07 |
| Arts, entertainment, and recreation | 15 | 1.79 |
| Other service activities | 113 | 13.45 |
| Unknown field of industry | 34 | 4.05 |

*Note.* ^1^categorized as industrial sector worker for analysis.
