# Supplementary material for: Drinking and Social Media Use Among Workers During COVID-19 Pandemic Restrictions: Five-Wave Longitudinal Study
Source: J Med Internet Res. 2021 Dec 2;23(12):e33125. doi: 10.2196/33125 (PMC8641700; doi:10.2196/33125)
Supplement: Multimedia Appendix 2 [file jmir_v23i12e33125_app2.docx]

**Table S1.** Descriptive statistics on use of social media platforms and associations to risky drinking in multi-level linear hybrid regression models

| **Use of general social media platforms** | **T1, %** | **T2, %** | **T3, %** | **T4, %** | **T5, %** | **B (within-person)** | **P** | **B (between-person)** | **P** |
| --- | --- | --- | --- | --- | --- | --- | --- | --- | --- |
| Facebook | 67.38 | 67.98 | 68.45 | 68.45 | 67.74 | 0.15 | 0.121 | 0.21 | 0.257 |
| YouTube | 66.31 | 63.93 | 65.83 | 64.05 | 66.55 | 0.07 | 0.147 | -0.22 | 0.287 |
| Instagram | 36.55 | 38.10 | 39.4 | 42.74 | 42.26 | -0.11 | 0.112 | 0.25 | 0.161 |
| Twitter | 12.38 | 12.62 | 14.05 | 14.76 | 14.76 | -0.09 | 0.346 | -0.15 | 0.576 |
| Pinterest | 10.48 | 9.29 | 11.19 | 11.19 | 11.9 | 0.09 | 0.361 | 0.04 | 0.883 |
| Tinder | 5.95 | 5.83 | 5.95 | 5.24 | 5.71 | 0.20 | 0.051 | 1.85 | <0.001 |
|  |  |  |  |  |  |  |  |  |  |
| **Use of social media platforms for work-purposes** |  |  |  |  |  |  |  |  |  |
| Linkedin | 11.79 | 9.52 | 10.00 | 9.05 | 9.40 | 0.07 | 0.371 | -0.50 | 0.067 |
| MS Teams | 8.45 | 12.50 | 23.45 | 32.98 | 38.21 | -0.04 | 0.459 | -0.43 | 0.062 |
| Yammer | 8.69 | 8.10 | 8.69 | 8.10 | 7.50 | 0.22 | 0.042 | -0.50 | 0.084 |
| Workplace by Facebook | 3.10 | 3.69 | 3.33 | 2.98 | 3.81 | -0.04 | 0.838 | -0.05 | 0.922 |
| Slack | 2.38 | 2.50 | 3.10 | 2.98 | 3.57 | 0.17 | 0.197 | -0.30 | 0.443 |
